# Differences in postoperative complications and elbow function between K-wire fixation of Gartland Wilkins type 2 and 3 supracondylar humeral fractures in children

**DOI:** 10.1371/journal.pone.0345622

**Published:** 2026-04-09

**Authors:** Stijn van Cruchten, Albert F. Pull ter Gunne, Edo J. Hekma, Victor A. de Ridder, Diederik P. J. Smeeing

**Affiliations:** 1 Department of Surgery, Rijnstate Hospital Arnhem, Arnhem, Netherlands; 2 Department of Surgery, Wilhemina Children’s Hospital Utrecht, Utrecht, Netherlands; Shuguang Hospital Affiliated to Shanghai University of Traditional Chinese Medicine, CHINA

## Abstract

**Background:**

Although the pathophysiology of Gartland Wilkins type 2 (GW 2) and 3 (GW 3) fractures is comparable, there are distinctive nuances regarding anatomical compromise and fracture stability. These differences may imply unique trajectories of functional rehabilitation, radiological outcomes and reduction-related complications, but consequential differences in treatment outcomes have not been studied thoroughly.

**Goal:**

To describe complications after K-wire fixation of supracondylar humeral fractures in children, and identify differences in postoperative outcomes between GW2 and GW3 fractures following K-wire fixation.

**Methods:**

A retrospective single-center cohort study was conducted. Children below 16 years of age that underwent K-wire fixation of a supracondylar fracture between January 2013 and October 2023 were identified. Patient characteristics, functional and radiological outcomes and postoperative complications were collected and compared between patients with GW 2 and GW 3 fractures.

**Results:**

A total of 178 patients were included in the analyses, of which 14.0% had a postoperative complication. The GW grade 3 group had a shorter time between trauma and surgery (p < 0.001), a longer time between surgery and K-wire removal (34.5 versus 31.7 days, p = 0.008) and a longer time between surgery and cast removal (34.9 versus 32.8 days, p = 0.017) than the GW grade 2 group. The GW-grade 3 group had a significantly lower rate of closed reduction than the GW-grade 2 group (48.4% versus 77.1%, p < 0.001). The GW3 group had a significantly higher rate of complications than the GW2 group (21.1% versus 6.0%, p = 0.005). This was mainly due to a higher rate of loss of reduction (0.0% versus 9.5%, p = 0.004). Rates of infection, nerve damage and chronic pain were also higher in the GW3 group, although not significant.

**Conclusion:**

In this cohort, 14.0% of all patients had a postoperative complication. GW3 fractures treated with K-wires were found to have significantly more complications than GW2 fractures. Also, the rate of loss of reduction was significantly higher in the GW3 group.

## Introduction

Supracondylar fractures of the humerus are common in children and account for more than half of pediatric elbow fractures [1]. The Gartland-Wilkins classification (GW) is a classification system used to assess the severity of the fracture and is based on dislocation, rotation and the state of the posterior cortex and functions as a cornerstone in guiding treatment decisions [2]. Already in 1948, Swenson et al. first described the treatment of supracondylar fractures of the humerus with K-wires [3]. Since then, this method has been extensively applied in daily practice due to its short fixation time and minimally invasive nature. The Dutch Guideline on elbow fractures recommends non-operative treatment for GW type 1 and 2a fractures and K-wire fixation following (open, or if possible closed) reduction for GW type 2b and 3 fractures [4]. Although the pathophysiology of GW2 and GW3 fractures is comparable, there are distinctive nuances regarding anatomical compromise and fracture stability [5–7]. These differences may imply unique trajectories of functional rehabilitation, radiological outcomes, and reduction-related complications; however, they have not yet been studied thoroughly [8,9]. Understanding potential divergent outcomes per fracture type can refine rehabilitation strategies and further improve patient-specific counseling. Therefore, the aim of this study was to describe complications after K-wire fixation of supracondylar humeral fractures in children in a single-center cohort and identify differences in postoperative outcomes between GW2 and GW3 fractures following K-wire fixation.

## Materials and methods

A retrospective cohort study was conducted in children with a supracondylar fracture who underwent K-wire fixation. For this report, we adhered to the Strengthening the Reporting of Observational Studies in Epidemiology (STROBE) guidelines [10]. A waiver from the Institional Review Board (IRB) and local approval were obtained (LHC number 2023–2323). Patient data was accessed as from October 5^th^, 2023, after approval of the IRB. Electronic hospital records were checked to ensure patients had no objection to the use of their data in scientific research. Patients’ data were analyzed pseudo-anonymously, and only the first author (SC) had access to information that could identify individual participants during or after data collection.

### Patient selection

The patient database of a level 2 trauma center, a teaching hospital in The Netherlands, was used. All individual patients who underwent K-wire fixation of a supracondylar fracture between January 2013 and October 2023 were identified. All patients below 16 years of age with a supracondylar fracture of the humerus GW type 2 and 3 fixated with K-wires were considered for inclusion in the study. Patients with preexisting bone diseases or neurological diseases and patients with a follow-up of less than 4 weeks were excluded from the analysis.

### Study variables

The patients’ medical records were screened to collect sex, age, height, and weight. Data concerning the affected side and trauma mechanism were extracted. To determine fracture type based on the GW classification, two authors (SC and DS) assessed all preoperative radiographs. During this process, no fractures were identified that retrospectively would have been more appropriately managed conservatively. It was noted whether the fracture consisted of more than two bone parts. In terms of treatment, the following variables were collected: time interval between trauma and treatment in days, open or closed reduction, fixation with crossed or lateral K-wires, number of K-wires, and adverse events. The time interval between surgery and K-wire removal, duration of postoperative cast immobilization, and total duration of follow-up were also extracted from the medical records.

### Outcome variables

All complications that occurred during and after treatment were collected from the medical records. Treatment of complications and the time between treatment and complication were also recorded. Complications were divided into infections, nerve damage, vascular damage, loss of reduction, loss of function, and ‘other’ complications. Infection was defined as pin tract infection, cutaneous infections that required antibiotic treatment or osteomyelitis. Nerve damage was documented as a complication when a postoperative neurological deficit was observed, but only if neural function was intact prior to the surgery and therefore the nerve injury was not of traumatic but iatrogenic nature. The severity of complications was assessed using the Clavien Dindo classification [11]. To determine loss of reduction, radiographs obtained intraoperatively and at 4-week follow-up were assessed and compared, in accordance with our institutional protocol for the management of these fractures. In addition, if clinical signs suggestive of possible loss of reduction were present, such as increasing pain, an earlier radiographic evaluation was performed. Baumann angles and lateral capitellohumeral angles (LCHA) were measured. The Baumann angle was defined as the angle formed by the humeral axis and a straight line through the epiphyseal plate of the capitulum on the frontal radiograph. The LCHA is the angle between the anterior humeral line and another line along the proximal border of the capitellar physis on the lateral radiograph. No existing studies identifying the mean clinically important difference (MCID) of the Baumann Angle and LCHA were found. Therefore, loss of reduction was defined as a loss of more than 10° in either Baumann angle or LCHA, and was only registered as a complication when there were remaining complaints and patients required extended follow up or subsequent treatment. Postoperative function was analysed by consultation of the follow up records. Function of the elbow joint was assessed by means of the Flynn’s criteria [12], in which a loss of function of more than 15° is defined as an unsatisfactory result. Loss of function was therefore defined as a loss of range of either flexion or extension of more than 15°. In daily practice, patients are discharged from follow-up after one to two months, often with a remaining limited function of the elbow, and are provided with exercise instructions or a referral to a physiotherapist. Although long-term elbow function has not been examined in these patients, their elbow function and range of motion are sufficient for them not to contact the outpatient clinic again. Therefore, a subanalysis was conducted in which the assumption was made that when patients were discharged from follow-up within two months and did not contact the outpatient clinic for remaining complaints, their functional outcome was satisfactory for the patient.

Other complications were defined as any other complications that occurred during treatment and follow-up.

### Statistical analysis

All statistical analyses were performed using IBM SPSS Statistics version 22.0.0.2 (IBM Corp., Armonk, NY). Outcome variables were compared between patients with Gartland Wilkins type 2 and type 3 fractures. Continuous variables were assessed for normality using the Shapiro–Wilk test. As several variables demonstrated significant deviation from a normal distribution, non-parametric analyses were performed for between-group comparisons. Continuous data are therefore presented as median and interquartile range (IQR), and differences between groups were analyzed using the Mann–Whitney U test. Chi-square tests and Fisher’s exact test for categorical variables and are presented as frequencies with percentages. A p-value of <0.05 was considered statistically significant.

## Results

### Patient selection and baseline characteristics

**Fig 1:**
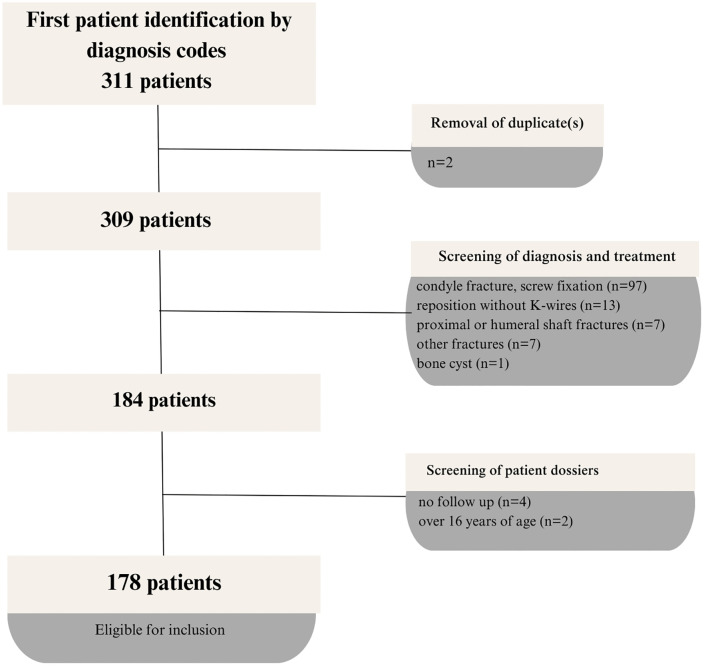
Flow diagram depicting the process of patient selection for inclusion in the analysis, of children with supracondylar fractures treated with K-wires.

A preliminary screening of treatment diagnosis codes identified a total of 311 children with elbow fractures surgically treated at Rijnstate Hospital in Arnhem, the Netherlands, between January 2013 and October 2023. After removing duplicates (n = 2), the remaining cases were examined in detail, leading to the exclusion of 125 patients with fracture types not intended for the study. Another six patients were excluded due to lack of follow-up or being over 16 years of age. Ultimately, 178 patients were included in this study and analyses (Fig 1). Table 1 presents the baseline characteristics of the total group, as well as the GW grade 2 (n = 83) and GW grade 3 (n = 95) subgroups. The GW grade 2 group had a lower mean BMI (15.3 versus 16.5, p = 0.017), a shorter time between trauma and surgery (p < 0.001), a shorter time between surgery and K-wire removal (31.7 versus 34.5 days, p = 0.008), and a shorter time between surgery and cast removal (32.8 versus 34.9 days, p = 0.017) compared to the GW grade 3 group. Also, the GW grade 2 group had a significantly higher rate of closed reduction than the GW grade 3 group (77.1% versus 48.4%, p < 0.001).

**Table 1:** Baseline characteristics of all included children with a supracondylar fracture, comparing data of patients with Gartland Wilkins type 2 and type 3 fractures.

Baseline characteristics	Total (n = 178)	GW type 2 (n = 83)	GW type 3 (n = 95)	p-value
Male (%)	84 (47.2)	41 (49.4)	43 (45.3)	0.581
Median age (IQR)	6 (3.00)	6 (3.00)	7 (3.00)	0.877
Median Length (IQR)	1.26 (0.19)	1.25 (0.17)	1.26 (0.20)	0.496
Median weight (IQR)	23 (10)	22.50 (11)	25.0 (13)	0.483
Median BMI (IQR)	14.88 (2.92)	14.65 (2.31)	15.94 (4.36)	0.017
Affected side, left (%)	112 (62.9)	56 (67.5)	56 (58.9)	0.246
Trauma Mechanism (%)				0.937
Sports/playground	125 (70.2)	59 (71.1)	66 (69.5)	
At home	14 (7.9)	6 (7.2)	8 (8.4)	
Traffic accident	18 (10.1)	7 (8.4)	11 (11.6)	
Non-accidental	0 (0.0)	0 (0.0)	0 (0.0)	
Not registered	19 (10.7)	10 (12.0)	9 (9.5)	
Time between trauma and treatment				**<0.001**
Same day (%)	104 (58.4)	36 (43.4)	68 (71.6)	
2 days (%)	65 (36.5)	40 (48.2)	25 (26.3)	
3 days (%)	5 (2.8)	3 (3.6)	2 (2.1)	
>3 days (%)	4 (2.2)	4 (4.8)	0 (0.0)	
Type of reduction (%)				**<0.001**
Closed	110 (61.8)	64 (77.1)	46 (48.4)	
Open	52 (29.2)	11 (13.3)	41 (43.2)	
Not registered	16 (9.0)	8 (9.6)	8 (8.4)	
Type of k-wire fixation (%)				1.000
Crossed	174 (97.8)	81 (97.6)	93 (97.9)	
Lateral	4 (2.2)	2 (2.4)	2 (2.1)	
Number of k-wires used				0.676
1 (%)	1 (0.6)	0 (0.0)	1 (1.0) 87 (91.6)	
2 (%)	164 (92.1)	77 (92.8)		
3 (%)	10 (5.6)	5 (6.0)	5 (5.3)	
4 (%)	3 (1.7)	1 (1.2)	2 (2.1)	
Median days until K-wire removal (IQR)	30.50 (10)	30.00 (8)	33.50 (12)	**0.008**
Median days of cast immobilisation (IQR)	31.00 (11)	32.8 (10)	34.9 (13)	**0.016**
Median months of follow up (IQR)	2.00 (1.6)	2.00 (1.5)	2.50 (2.5)	0.136

### Complications

The GW2 group had a significantly lower rate of complications than the GW3 group (6.0% versus 21.1%, p = 0.005), mainly due to a higher rate of loss of reduction (0.0% versus 9.5%, p = 0.004). The rate of loss of reduction was similar between patients who underwent closed and open reduction (7.3% versus 5.8%, p = 1.000). The rates of infections, nerve damage, loss of function, and chronic pain did not differ significantly between groups (Table 2). Nerve damage occurred in three patients (3.6%) in the GW2 group and six patients (6.3%) in the GW3 group (p = 0.506), consisting of five ulnar neuropraxias, two median neuropraxias, and two radial neuropraxias. All of these patients treated with crossed and not lateral K-wires fixation. Also, there was no significant difference in the rate of nerve damage and the type of reduction (closed 4.5% versus open 7.7%, p = 0.470). All neuropraxias was treated with a “wait and see” policy, and all patients showed complete recovery during follow-up. Peroperative vascular damage did not occur in this cohort. In both groups, 80.0% of registered complications were graded Clavien-Dindo 1 or 2, whereas 20.0% were graded Clavien-Dindo 3–5 (p = 1.000). In the GW2 group, four out of five patients (80%) were treated conservatively (antibiotics, wait and see, physiotherapy), while one patient (20%) received a second operation. In the GW3 group, 16 patients (80%) were treated conservatively, three patients (15%) received a second operation, and one patient (5%) sought a second opinion (p = 0.744). In three out of five patients (60%) in the GW2 group, there was full return of function at last follow-up, compared to 13 out of 20 patients in the GW3 group (p = 1.000). For the other patients (GW2 n = 2 versus GW3 n = 7), remaining complaints were documented and the current patient condition could not be retrieved from the electronic patient records.

**Table 2:** Comparison between the type, severity and treatment of complications in the Gartland Wilkins 2 and Gartland Wilkins 3 groups after severity of complications is graded using the Clavien Dindo classification [11].

Complications	Total (n = 178)	GW 2 (n = 83)	GW 3 (n = 95)	p-value
Infection, n (%)	2 (1.1)	0 (0.0)	2 (2.1)	0.499
Loss of reduction, n (%)	9 (5.1)	0 (0.0)	9 (9.5)	0.004
Nerve damage, n (%)	9 (5.1)	3 (3.6)	6 (6.3)	0.506
Loss of function, n (%)	4 (2.2)	2 (2.4)	2 (2.1)	0.624
Chronic pain, n (%)	1 (0.6)	0 (0.0)	1 (1.1)	1.000
Total	25 (14.0)	5 (6.0)	20 (21.1)	0.005
Severity of complications	**Total (n = 25)**	**GW 2 (n = 5)**	**GW 3 (n = 20)**	**p-value**
Clavien dindo 1–2, n (%)	20 (80.0)	4 (80.0)	16 (80.0)	1.000
Clavien dindo 3–5, n (%)	5 (20.0)	1 (20.0)	4 (20.0)	
Treatment of complications	**Total (n = 25)**	**GW 2 (n = 5)**	**GW 3 (n = 20)**	**p-value** 0.744
Wait and see/physiotherapy, n (%)	18 (72.0)	4 (80.0)	14 (70.0)	
Antibiotics, n (%)	2 (8.0)	0 (0.0)	2 (10.0)	
Surgery, n (%)	4 (16.0)	1 (20.0)	3 (15.0)	
Second opinion, n (%)	1 (4.0)	0 (0.0)	1 (5.0)	

### Functional and radiological outcomes

Postoperative function was measured using Flynn’s criteria, with results presented in Table 3. In a subanalysis where elbow function in all patients with follow-up of less than two months was graded as good, GW 2 and 3 patients had similar satisfactory to good outcomes (85.2% versus 88.6%, p = 0.838). The GW2 group had a significantly higher mean postoperative LCHA (48.92° versus 45.28°, p = 0.026). There were no other significant differences in preoperative and postoperative Baumann angle and LCHA (Table 4). Mean gradual changes in Baumann angle and LCHA were comparable, with similar rates of loss of reduction (4.8% versus 9.5%, p = 0.264) (Table 5).

**Table 3:** Comparison of loss of elbow function between the Gartland Wilkins 2 and Gartland Wilkins 3 groups, loss of elbow function was graded as good, satisfactory or unsatisfactory, based on Flynn’s criteria [12].

Loss of function by Flynn’s criteria	GW 2 (n = 83)	GW 3 (n = 95)	p-value
Good (0°), n (%)	39 (48.1)	53 (57.0)	0.324
Satisfactory (<15°), n (%)	23 (28.4)	26 (28.0)	
Unsatisfactory (>15°), n (%)	19 (23.5)	14 (15.0)	
**Loss of function with function of patients discharged from follow up within 2 months graded as good**			
Good (0°), n (%)	54 (64.2)	63 (65.6)	0.838
Satisfactory (<15°), n (%)	17 (21.0)	21 (22.6)	
Unsatisfactory (>15°), n (%)	12 (14.8)	11 (11.8)	

**Table 4:** Comparison of Baumann angle and Lateral Capitellohumeral Angle in grades, between the Gartland Wilkins 2 and Gartland Wilkins 3 groups.

Radiological angles	Peroperative			Postoperative		
	GW 2	GW 3	p-value	GW 2	GW 3	p-value
Baumann angle, median (IQR)	70.08 (8)	71 (6)	0.517	68 (6)	70 (9)	0.443
LCHA, median (IQR)	49 (10)	44 (10)	0.060	50 (12)	45 (12)	**0.026**

**Table 5:** Changes in Baumann angle and Lateral Capitellohumeral Angle (LCHA) between peroperative radiographs and radiographs 1 month after surgery.

Change in radiological angles after surgery	GW 2 (n = 83)	GW 3 (n = 95)	*p-*value
Δ Baumann angle	4.35 (3.384)	4.79 (3.637)	0.525
Δ LCHA	4.39 (2.418)	4.62 (3.676)	0.832
Patients with loss of reduction (>10°)	4 (4.8)	9 (9.5)	0.264

## Discussion

This study compared functional outcomes and complications following operative treatment using K-wire fixation for GW2 and GW3 supracondylar fractures of the humerus in patients under 16 years of age. Within this cohort, 14.0% of patients experienced postoperative complications. Fixation of GW3 fractures was associated with significantly higher complication rates compared to GW2 fractures, particularly regarding loss of reduction. However, there were no significant differences in complication severity, and most complications were manageable conservatively. Postoperative range of elbow motion did not significantly differ between the groups.

These findings suggest that clinically, this underscores the importance of thorough preoperative counseling for GW3 fractures, informing caregivers about the increased likelihood of radiographic instability, potential longer recovery trajectories, and, in some cases, the possibility of secondary intervention. In addition, clinicians should maintain a low threshold for obtaining follow-up radiographs in GW3 patients when there is are any clinical signs of instability, so that potential loss of reduction can be detected and managed early.

Two prior studies examining fracture type’s influence on complications concurred with our findings. Korner et al. observed a higher incidence of complications in patients with GW3 fractures (87.5%) compared to those without complications (30.3%, p = 0.027), without analyzing severity differences between fracture types [13]. Similarly, Osateerakun et al. reported a significantly higher complication risk in GW3 fractures (38.5% versus 6.1%, p = 0.01) [14]. The observed complication rate in our study falls within the range reported in existing literature (0% to 24.4%) [14–18], possibly attributable to variations in complication definitions, reporting practices among institutions and general level of healthcare and peroperative infection prevention.

Loss of reduction outcomes after K-wire treatment of GW2 and GW3 fractures were investigated in two studies: Sangkomkamhang et al. found a higher risk of loss of reduction in GW3 fractures [19], whereas Balakumar et al. did not find GW3 fractures independent for postoperative loss of reduction (p = 0.590) [20]. Although firm conclusions regarding the differences in reported findings cannot be drawn given the limited available information, one possible explanation is that these studies included a higher proportion of patients treated exclusively with laterally placed K-wires. Other potentially influential factors may include surgical experience, choice of implant (e.g., K-wire diameter), and the use of and adherence to postoperative protocols. The overall loss of reduction rate in our cohort was 5.1%, consistent with literature rates ranging from 0% to 13.4%, although varying definitions of loss of reduction were used [1,18,21–25]. The clinical significance of these findings, particularly regarding Baumann’s angle and Lateral Condylar Humeral Angle (LCHA), remains unclear due to the absence of established minimal clinically important differences. Also, in this study a significant difference between postoperative LCHA in the GW2 group and GW3 group was found, but this difference is small (3.04°) and, as both values lie within the reported range of normal LCHA [26], it is likely to be clinically irrelevant. The national guideline advises follow-up radiographs of the elbow 7–10 days after surgery, to discover loss of reduction timely and consider possible revision of the osteosynthesis [4].

Regarding functional outcomes, conflicting evidence exists between GW2 and GW3 fractures treated with K-wires. Poulios et al. found no significant differences in satisfactory range of motion (p = 0.606) [8], whereas Sheikdon et al. identified GW1 fractures, not GW2 or GW3 fractures, as significantly associated with unsatisfactory outcomes (p = 0.013) [9]. Flynn’s grading system, widely used to assess functional outcomes post-fixation, reports satisfactory outcomes varying from 80.0% to 96.4% across different studies, albeit with varying follow-up durations [15,16,24,27–29]. However, duration of follow up varies between studies. Although the findings of this cohort are in line with existing literature, the retrospective study design led to a high rate of patients to be discharged before full return of function. Only three patients had to undergo another surgical procedure because of loss of function.

The reported rate of iatrogenic nerve palsy after K-wire fixation of supracondylar fractures in current literature ranges from 0.0% to 6.9% [6,21,24,29–31]. Preoperative nerve palsy as a consequence of initial trauma, were excluded from the reported incidences. In the cohort of this study, in 5.1% of patients an iatrogenic nerve palsy occurred. The differences in the reported rates may possibly be caused by a difference in peroperative attendance to nerve perseverance and protection. Studies specifically investigating nerve damage occurrence between fixation of GW2 and GW3 fractures were not found.

Vascular injury was not present in this cohort. Vascular injury, mostly of the brachial artery, is a serious complication of supracondylar fractures. Although there are reports of peroperative brachial injury during K-wire pinning [32], vascular damage is mostly described as due to the initial trauma rather than to the fixation of the fracture [23,33].

Patients with GW3 fractures underwent surgery sooner after trauma and had longer periods of K-wire and cast immobilization, likely due to the inherent instability of these fractures. Literature on the influence of timing of surgery on postoperative outcomes remains inconclusive, with most studies reporting no significant differences in complication rates between early and delayed surgeries for GW3 fractures [18,34–37]. However, a single study suggested delayed surgery (>2 days post-trauma) may negatively impact outcomes [9]. No published studies comparing different durations of K-wire fixation and cast immobilization were identified, although a radiological union within 3–4 weeks is generally described in current literature [38,39].

Strengths of our study include a relatively large cohort of 178 fractures and comprehensive assessment of various outcomes. Limitations include the retrospective design of this study. GW3 fractures always require operative management, whereas GW2 fractures may be treated either operatively or non-operatively depending on fracture stability. Due to the retrospective nature of the study, the exact indications for surgical intervention could not always be retrieved from the medical records. However, during radiographic review, no fractures were identified that demonstrated clear features warranting conservative treatment instead of K-wire fixation. Also, no intra- or inter observer reliability analysis was performed regarding the retrospective radiological classification of fractures, which could also be considered a limitation of this study. The retrospective design also led to varied follow-up durations, affecting the complete assessment of postoperative elbow function in discharged patients. During the study period, all procedures were performed by designated trauma or orthopedic surgeons with substantial experience in pediatric upper extremity fracture management. Despite surgical management followed institutional standards for K-wire fixation, we cannot completely exclude inter-surgeon variability.

## Conclusion

The data of this cohort showed that 14.0% of children had a postoperative complication after K-wire fixation of a supracondylar fracture. Loss of reduction and nerve damage occurred most frequently, both in 5.1%. There was a significant correlation between fracture grade and the total rate of complications: GW3 fractures treated with K-wires were found to have to significantly more complications than GW2 fractures. Also, the rate of loss of reduction was significantly higher in the GW3 group. These findings may be used to refine preoperative counseling and postoperative instructions and rehabilitation strategies.

## Supporting information

S1 Data Dataset. Raw data underlying the results.
(XLSX)
